# X-linked spinal and bulbar muscular atrophy (Kennedy’s disease): the first case described in the Brazilian Amazon

**DOI:** 10.1590/S1679-45082018RC4011

**Published:** 2018-05-25

**Authors:** Camila Nascimento Alves, Tiago Kiyoshi Kitabayashi Braga, Danusa Neves Somensi, Bruno Sérgio Vilhena do Nascimento, José Antônio Santos de Lima, Satomi Fujihara

**Affiliations:** 1 Hospital Ophir Loyola, Belém, PA, Brazil.; 2 Universidade do Estado do Pará, Belém, PA, Brazil.

**Keywords:** Bulbo-spinal atrophy, X-Linked, Kennedy disease, Motor neurons, Motor neuron disease, Medulla oblongata, Amazonian ecosystem, Brazil, Case reports, Atrofia bulboespinhal ligada ao X, Síndrome de Kennedy, Neurônios motores, Doença dos neurônios motores, Bulbo, Amazônia, Brasil, Relatos de casos

## Abstract

The X-linked spinal and bulbar muscular atrophy (Kennedy’s disease) is a rare X-linked, recessive, lower motor neuron disease, characterized by weakness, atrophy, and fasciculations of the appendicular and bulbar muscle. The disease is caused by an expansion of the CAG repetition in the androgen receptor gene. Patients with Kennedy’s disease have more than 39 CAG repetitions. We report a case of 57-year-old man, resident of Monte Dourado (PA, Brazil) who complained of brachiocrural paresis evolving for 3 years along with fasciculations and tremors of extremities. In addition, he also developed dysarthria, dysphagia, and sexual dysfunction. The patient clinical picture included gait impairment, global hyporeflexia, proximal muscle atrophy of upper limbs, deviation of the uvula to right during phonation and tongue atrophy with fasciculations. The patient reported that about 30 years ago he had undergone gynecomastia surgery. His electroneuromyography suggested spinal muscular atrophy, and nuclear magnetic resonance imaging showed tapering of the cervical and thoracic spinal cord. Patient’s creatine kinase level was elevated. In view of the findings, an exam was requested to investigate Kennedy’s disease. The exam identified 46 CAG repetitions in the androgen receptor gene, which confirmed the diagnostic suspicion. This was the first case of Kennedy’s disease diagnosed and described in the Brazilian Amazon. To our knowledge only other four papers were published on this disease in Brazilian patients. A brief review is also provided on etiopathogenic, clinical and diagnostic aspects.

## RESUMO

A atrofia muscular bulboespinhal ligada ao cromossomo X (doença de Kennedy) é uma rara doença de neurônio motor inferior, recessiva, ligada ao X, e caracterizada por fraqueza, atrofia e fasciculações da musculatura apendicular e bulbar. É causada por uma expansão da repetição CAG no gene do receptor de androgênio. Pacientes com doença de Kennedy apresentam mais de 39 repetições CAG. O paciente deste relato era do sexo masculino, 57 anos, morador de Monte Dourado (PA, Brasil), com queixa de paresia braquiocrural há 3 anos, acompanhada de fasciculações e tremores de extremidades. Em seguida, ele desenvolveu disartria, disfagia e disfunção sexual. Também apresentava comprometimento da marcha, hiporreflexia global, atrofia muscular proximal dos membros superiores, desvio da úvula para direita à fonação e atrofia de língua com fasciculações. Foi realizada cirurgia para tratamento de ginecomastia há 30 anos. A eletroneuromiografia sugeriu quadro de atrofia muscular espinhal. Imagens de ressonância magnética demonstraram afilamento da medula espinhal cervical e torácica. A creatina quinase estava elevada. Diante dos achados, solicitou-se investigação para doença de Kennedy, e foram identificadas 46 repetições CAG no gene do receptor de androgênio, o que confirmou a suspeita diagnóstica. Este foi o primeiro caso de doença de Kennedy diagnosticado e descrito na Amazônia brasileira. Existem, além deste relato, apenas outros quatro trabalhos publicados sobre a doença em pacientes do Brasil. Também realizamos breve revisão de aspectos etiopatogênicos, clínicos e diagnósticos.

## INTRODUCTION

Spinal and bulbar muscular atrophy (SBMA) or Kennedy’s disease is a rare lower motor neuron disease, X-linked recessive inheritance, characterized by weakness, atrophy and appendicular or bulbar muscles fasciculations. This disease is caused by a cytosine-adenine-guanine (CAG) repeat expansion in exon 1 of androgen receptor gene located in X-chromosome (Xq11-q12). This was the first repeat expansion mutation identified.^(^ ^1^ ^,^ ^2^ ^)^

Cytosine-adenine-guanine repetition codifies a polyglutamine tract that presents 10 to 36 residues in normal individuals whereas patients with SBMA have more than 39 residues.^(^ ^3^ ^)^

Kennedy’s disease prevalence ranged among studies. Kaimen-Maciel et al.,^(^ ^4^ ^)^ reported a prevalence around 1 case within 50,000 men, and La Spada et al.,^(^ ^5^ ^)^ 1 case within 300,000. Currently, no specific treatment exists for SBMA. The approach used includes supporting treatment such as physiotherapy and rehabilitation (including the use of braces and walkers), gynecomastia surgery when necessary, prevention of secondary complications (mainly those resulted from bulbar weakness such as pneumonia and asphyxia due to these diseases fatality rate), annual follow-up of muscle strength and annual follow-up with pulmonary-function testing in advanced cases.^(^ ^5^ ^)^ In addition, exercise can also benefit patients.^(^ ^1^ ^)^

In Brazil, few reports have described patients with SBMA. To our knowledge a total of 16 patients have been diagnosed so far, 2 by Seelfeld et al.,^(^ ^6^ ^)^ 3 by Kaimen-Maciel et al.,^(^ ^4^ ^)^ 1 by Kouyoumdijan et al.,^(^ ^7^ ^)^ and 10 by Dias et al.,^(^ ^2^ ^)^ We report the first case of SBMA diagnosed and described in the Brazilian Amazon.

## CASE REPORT

A 57-year-old man, resident of Monte Dourado (PA, Brazil), was referred to neurology assessment in the city of Belém (PA, Brazil) because of brachiocrural paresis, evolving for 3 years. Symptoms appeared in patient’s lower limbs. After 1 year, his upper limbs also presented paresis with fasciculation and tremor in extremities. Later, the patient developed dysarthria, dysphagia and sexual dysfunction. The patient had undergone surgical correction of gynecomastia 30 years ago.

His neurological exams showed compromised gait, incapability of walk on tiptoes, proximal muscular atrophy of upper limbs, and fasciculations in upper and lower limbs. Triceps, biceps, brachioradialis, pronator and patellar reflexes presented bilateral hyporeflexia (grade 1 in deep tendon reflexes grading scale). Right and left Achilles reflex were abolished (grade 0). In the Medical Research Council (MRC) scale, the patient had muscle strength of 4 in the bilateral lower limbs and proximal portion of upper limbs.

**Figure 1:**
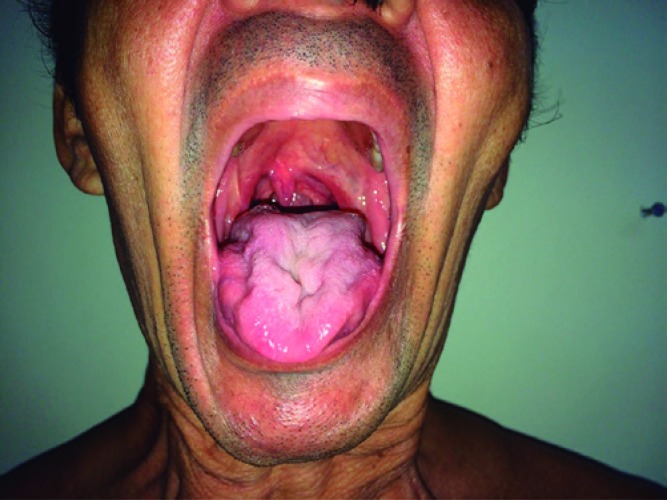
Tongue atrophy and deviation of uvula to right

**Figure 2:**
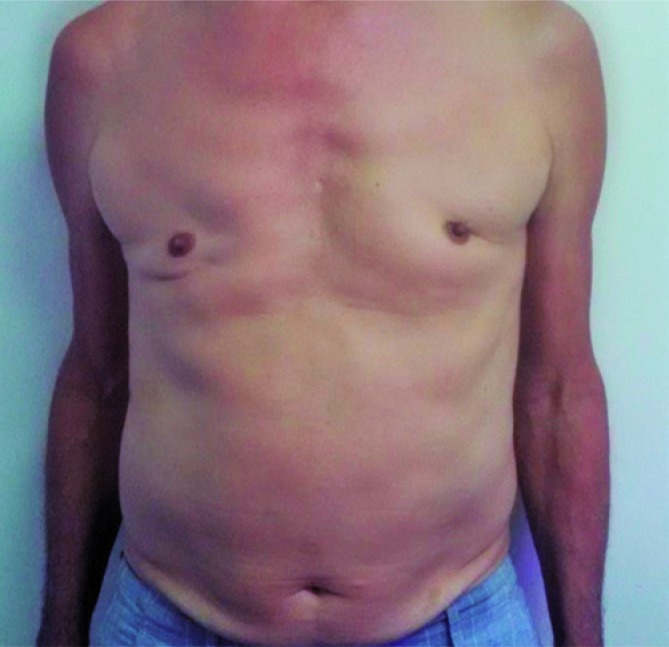
Surgical scars as a result of gynecomastia correction

We observed compromised bulbar because of deviation of the uvula to right due to phonation and tongue atrophy with fasciculations (Figure 1). He also had surgical scars as a result of the gynecomastia surgery (Figure 2).

In January 2015, nuclear magnetic resonance (NMR) images showed thinning of cervical and thoracic spine cord with discrete change in signal in C4, C5 and C6 without acquisition the image using contrast media and of unspecific aspect.

In electromyography, diffused fasciculations in upper limbs occurred in resting, notably in triceps, extensor digitorium and first dorsal interosseous of the hand. In his lower limbs, we observed fasciculations in tibialis anterior. The muscle activity was globally rarefied and showed few motor units with increased duration and amplitude. A neuroconduction study in upper limbs was performed and revealed a inexcitable sensitive conduction and a motor conduction showing greater latencies of the median nerve bilaterally.

Creatinophosphokinase (CPK) was high at two times, with the values: 1757U/I in June 2015 and 1638U/I in December 2015.

Based on findings, a genetic test was requested to investigate number of CAG repetition in AR gene by reaction in polymerase chain reaction (PCR). The exam was performed at the Human Genome and Stem Cell Research Center of the *Universidade de São Paulo*. Forty-six CAG repetitions were identified, which confirmed diagnosis of SBMA.

The patient had 10 brothers and 6 sisters. Of them, 2 of his brothers had symptoms such as difficulties for walking and climbing stairs. However, only 2 of 10 brothers underwent screening for SBMA. One of them was diagnosed with the disease in January/2017 by a genetic test (44 CAG repetitions). The patient’s brother who were diagnosed had loss of proximal muscle strength in lower limbs and gynecomastia. Patient’s other siblings did not undergo the investigation due to finance issues and because they lived far away from large urban centers.

## DISCUSSION

The AR is an intracellular receptor. In absence of ligand, it is located in cytoplasm in complex with heat shock proteins (HSP). In the presence of ligand, testosterone or dihydrotestosterone, the receptor is dissociated from these proteins and enters on nucleus, in which interacts with other nuclear proteins and binds as a dimer to recognition sequences in the DNA of target genes. For this reason, AR works as transcription factor depending on ligand that change the target gene expression.^(^ ^1^ ^)^

We conducted a literature review on cases of SBMA described in Brazil. We searched “PubMed”, “BIREME” and “SciELO” using the keywords “Kennedy’s Disease” or “Spinal and Bulbar Muscular Atrophy” or “Bulbospinal Muscular Atrophy” or “Bulbo Spinal Atrophy”. In “PubMed” and “BIREME”, the country of authors affiliation (Brazil or Brasil) was used as a filter. The searched retrieved 4 Brazilian articles that described 16 patients with SBMA (Table 1).

**Table 1:** Cases of Kennedy’s disease described in Brazil

Authors	Number of cases	State – Region of the country
Dias et al.^(^ ^2^ ^)^	10	Paraná – South
Kaimen-Maciel et al.^(^ ^4^ ^)^	4*	Paraná – South
Seefeld et al.^(^ ^6^ ^)^	2	Paraná – South
Kouyoumdjan et al.^(^ ^7^ ^)^	1	São Paulo – Southeast
Our case	1	Pará – North

* Three cases and one asymptomatic female carrier.

Our case was the first diagnosed and described in the Brazilian Amazon. Still, few cases of SBMA have been described in Brazil. It is important to mention that, although Seelfeld et al.,^(^ ^6^ ^)^ and Dias et al.,^(^ ^2^ ^)^ are from the same institution, they presented different casuistic.

By analyzing Brazilian cases, we observed that mean age of onset of symptoms was 35.5 years, and ranged from 14 to 49 years, among 16 patients. Of initial symptoms reported, the dysphonia occurred in one individual,^(^ ^7^ ^)^ dysphagia and dysarthria were observed in another,^(^ ^4^ ^)^ cramp in one,^(^ ^6^ ^)^ augmentation of the breast volume in one^(^ ^4^ ^)^ and muscular weakness in three patients.^(^ ^4^ ^,^ ^6^ ^)^ Dias et al.,^(^ ^2^ ^)^ did not provide details about initial symptoms of ten patients they diagnosed; however when the study was conducted, all patients reported appendicular weakness, mainly proximal, associated with bulbar symptoms; the postural tremor in hands was the most common tremor type. Gynecomastia was observed in 14 of the 16 patients. Only 2 of studies reported number of CAG repetitions,^(^ ^2^ ^,^ ^7^ ^)^ the smaller number of repetitions was 41 and greater was 53. Kaimen-Maciel et al.,^(^ ^4^ ^)^ used the number of pairs of AR base for the diagnosis. In the Seelfeld et al.,^(^ ^6^ ^)^ paper, the heredogram of one patient showed existence of other 10 cases with similar symptoms in the family. Kaimen-Maciel et al.,^(^ ^4^ ^)^ presented heredogram that, in addition to three cases described, there were seven men who had symptoms of SBMA (one of them who had already died), but they were not investigated with genetic testing (Table 2).

**Table 2:** Characteristics of 16 cases of Kennedy’s disease described in Brazil

Characteristics	Results
Mean age of onset of symptoms	35.5 years (14-49)
Mean of CAG repetitions*	46.72 repetitions (41-53)
Initial symptoms described†,‡	Muscle weakness: 3 individuals Gynecomastia: 1 individual Cramps: 1 individual Dysphagia e dysarthria: 1 individual Dysphonia: 1 individual
Gynecomastia	14 of 16 patients
Affected family members§	Seelfed et al.:^(^ ^6^ ^)^ 10 men with symptoms of SBMA Kaimen-Maciel et al.:^(^ ^4^ ^)^ 7 men with symptoms of SBMA (one was dead)

* Only studies by Dias et al.^(^ ^2^ ^)^ and Kouyoumdjan et al.,^(^ ^7^ ^)^ were considered because they reported number of CAG repetitions. The article by Seefeld et al.,^(^ ^6^ ^)^ did not include number of repetitions. Kaimen-Maciel et al.,^(^ ^4^ ^)^ used number of base pairs;

† the study by Dias et al.,^(^ ^2^ ^)^ was not included because it does not provide details on initial symptom of patients;

‡ Kaimen-Maciel et al.,^(^ ^4^ ^)^ described one of the initial symptoms of one patient with “global disability”. This term was not considered because of its imprecise description;

§ only the study by Seelfed et al.,^(^ ^6^ ^)^ and Kaimen-Maciel et al.,^(^ ^4^ ^)^ reported affected family members.

The polyglutamine expansion, determined by increase of CAG repetitions, results in both loss and gain of AR function. The loss of function is evident in the gynecomastia, reduction of fertility, muscle and neuronal degeneration; considering that androgens are trophic for motor neurons and anabolic for muscles. The gain of AR function would be acquired toxicity by mutant protein against neuron and muscle, therefore, explaining in a satisfactory manner the clinical picture, because other conditions that occur along with androgen insensitivity syndrome do not present motor manifestations.^(^ ^1^ ^)^

Atsuta et al.,^(^ ^8^ ^)^ observed that hand tremors was the earliest symptom to occur, mean age of occurrence was 33 years. Other symptoms that followed were muscle weakness mainly in lower limbs (44 years), need to use a handrail to climb stairs (49 years), dysarthria (50 years), dysphagia (54 years), need to use a walking stick (59 years), and use of wheelchair (61 years). Of 223 patients, 15 died – they were aged, on average at 65 years. Aspiration pneumonia was the most common cause of death (8 of the 15 patients died because of this problem).

In the study by Fratta et al.,^(^ ^9^ ^)^ patients developed erectile dysfunction between 50 and 60 years of age, mean age of patients who died was 79 years. The most common initial symptom was weakness in lower limbs that affected 86.7% of patients. During disease evolution, 58.7% of patients developed sensorial symptoms and 73.9% gynecomastia. Onset of symptoms often occurred in adult life, on average at 43.4 years. In a review, age of SBMA onset ranged from 4 to 76 years.^(^ ^10^ ^)^

Dias et al.,^(^ ^2^ ^)^ observed that the tremor was seen in 8 of 10 patients with SBMA who were evaluated and had similar characteristics of essential tremor.

In relation to neuroconduction, the reduction of sensory nerve action potentials (SNAP) is a common characteristic in SBMA, and compound muscle action potential (CMAP) of median nerve can be altered in 40% of patients.^(^ ^3^ ^,^ ^7^ ^)^

In electromyography, neurologic chronic changes are often more evident, with augmented motor unit potentials and reduced recruiting.^(^ ^3^ ^)^

Sperfeld et al.,^(^ ^11^ ^)^ showed significant atrophy of the cervical and thoracic spinal cord in patients with SBMA. However, the authors did not observe signal changes in magnetic resonance imaging of the central nervous system, and such changes were considered infrequent in motor neuron disease.

Querin et al.,^(^ ^12^ ^)^ observed high levels of creatine kinase (CK) in 94% of patients. Serum elevation of CK and myopathic changes found in biopsies of muscles suggest the possible existence of subjacent myopathy in SBMA.

Studies already observed significant correlation between CAG repetitions length and age in the disease onset. However, there is no correlation between repetitions length and disease evolve.^(^ ^8^ ^,^ ^9^ ^)^ Atsuta et al.,^(^ ^8^ ^)^ found one association between number of repetitions and age that patients started to present hand tremors, muscle weakness, dysarthria, dysphagia and patient’s age at death. However, no association was found between CAG length and evolution time between muscle weakness beginning and death.

Finsterer et al.,^(^ ^10^ ^)^ observed that many studies found a relationship between motor symptoms onset and CAG repetition length. However, when non-motor symptoms are considered, there is no relationship with number of repetitions.

Grunseich et al.,^(^ ^13^ ^)^ described a SBMA individual with a greater number of CAG repetitions (68 repetitions). Their patient had a congenital abnormality of the penis (chordee) that was corrected when he was 7-year-old, he also had testicular atrophy and difficult of ejaculation. When the patient was 16-year-old, he developed gynecomastia. When he turned 18-year-old, he began to loss muscular strength in proximal portion of lower limbs, to feel fatigue after exercise, fasciculation, cramps and tremors. In addition, the patient had sudomotor dysfunction. When the patient was 29 years, he underwent the genetic test and the diagnosed was confirmed.

In Huntington’s disease reduced penetrance is well established that occurs when 36 to 41 CAG repetitions are seen.^(^ ^14^ ^)^ However, in SBMA, reduced penetrance is not well defined. Spada et al.,^(^ ^5^ ^)^ ended up dividing alleles of SBMA in some categories: normal allele would have 34 or less repetitions, alleles with complete penetrance would have 38 or more CAG repetitions.

Still, there are alleles with reduced penetrance,^(^ ^5^ ^)^ which was suggested based on Kuhlenbäumer et al.,^(^ ^14^ ^)^ study. These authors reported a case of 86-year-old asymptomatic woman with 37/51 CAG repetitions, and her son, a 46-year-old asymptomatic man with 37 repetitions. Therefore, two possibilities were proposed: the first one would be a more precisely limit between normal alleles (up to 37 CAG) and pathological alleles (from 38 CAG), the second one is that 37 CAG repetitions would lead to reduced penetrance of the disease, therefore, leading to SBMA in a later age – so late that most of patients would die before the disease become evident. However, this puzzle can be solved only after follow-up of more individuals within the same level of repetitions length.^(^ ^14^ ^)^

Spinal and bulbar muscular atrophy is often confused with amyotrophic lateral sclerosis (ALS), about one in 25 individuals diagnosed with ALS actually has SBMA. For this reason, ALS is an important differential diagnosis. Differential diagnosis can be done with clinical history and physical exam. We must remember that ALS affects both upper and lower neurons, therefore, it is expected to exist hyperreflexia and spasticity, facts that do not occur in SBMA. Individuals with ALS also present a large group of affected muscles and the disease has a faster progress. Of note is that SBMA presents androgen insensibility, therefore gynecomastia is often found in men with SBMA.^(^ ^5^ ^)^ In addition, history of male family members affected by the disease favor the diagnosis of SBMA.

The current understanding of this disease is that mutant protein becomes toxic in presence of ligand (testosterone or dyhidrotestosterone). Protective mechanisms include heat shock response, ubiquitin-proteasome pathway and autophagy.^(^ ^1^ ^)^ Some years ago studies involving humans tested leuprorelin and dutasteride (antiandrogenic therapies), but they did not show useful results. Currently, many therapies are under testing in animal models.^(^ ^5^ ^)^

Fischbeck et al.,^(^ ^1^ ^)^ reported a number of molecular targets in which therapy was efficient in transgenic mice: enhancing the heat shock response through inhibition of Heat Shock Protein 90 (HSP90) with use of geldanamycin derivatives, 17-dimethylaminoethylamino-17-demethoxy-geldanamycin (17-DMAG) and allylamino-17-demethoxygeldanamycin (17-AAG); increasing of AR degradation and activation of protection pathways with curcumin derivatives, 5-hydroxy-1,7-bis (3,4-dimethoxyphenyl)-1,4,6-heptatrien-3-one (ASC-J9) and (1E,4Z,6E)-4-(cyclobutylmethyl)-1,7-bis (3,4-dimethoxyphenyl)-5-hydroxyhepta-1,4,6-trien-3-one (ASC-JM17); inhibition of CGRP-JNK (calcitonin gene-related peptide α - c-Jun N-terminal kinase) signaling with naratriptan, rescuing of mitochondrial function through peroxisome proliferator-activated receptor gamma (PPARγ) with pioglitazone.

Other therapy that presented efficacy in mice was the use of insulin-like growth factor 1 (IGF-1) that activates protein Akt and causes phosphorylation of AR mutant.^(^ ^1^ ^,^ ^5^ ^)^ Still, therapeutic strategies to reduce disease gene expression with miRNA and oligonucleotides have had promising preclinical results and they are close to clinical application.
